# Association of Onset-to-Treatment Time With Discharge Destination, Mortality, and Complications Among Patients With Aneurysmal Subarachnoid Hemorrhage

**DOI:** 10.1001/jamanetworkopen.2021.44039

**Published:** 2022-01-21

**Authors:** Marie-Jeanne Buscot, Ronil V. Chandra, Julian Mainguard, Linda Nichols, Leigh Blizzard, Christine Stirling, Karen Smith, Leon Lai, Hamed Asadi, Jens Froelich, Mathew J. Reeves, Nova Thani, Amanda Thrift, Seana Gall

**Affiliations:** 1 Menzies Institute for Medical Research, University of Tasmania, Hobart, Tasmania, Australia; 2 NeuroInterventional Radiology, Monash Health, Melbourne, Victoria, Australia; 3 School of Clinical Sciences Monash Health, Monash University, Melbourne, Victoria, Australia; 4 School of Nursing, University of Tasmania, Hobart, Tasmania, Australia; 5 Ambulance Victoria, Melbourne, Victoria, Australia; 6 Department of Neurosurgery, Monash Health, Melbourne, Victoria, Australia; 7 NeuroInterventional Radiology, Royal Hobart Hospital, Hobart, Tasmania, Australia; 8 Department of Epidemiology, Michigan State University, East Lansing; 9 Department of Neurosurgery, Royal Hobart Hospital, Hobart, Tasmania, Australia

## Abstract

**Importance:**

Rapid access to specialized care is recommended to improve outcomes after aneurysmal subarachnoid hemorrhage (SAH), but understanding of the optimal onset-to-treatment time for aneurysmal SAH is limited.

**Objective:**

To assess the optimal onset-to-treatment time for aneurysmal SAH that maximized patient outcomes after surgery.

**Design, Setting, and Participants:**

This cohort study assessed 575 retrospectively identified cases of first-ever aneurysmal SAH occurring within the referral networks of 2 major tertiary Australian hospitals from January 1, 2010, to December 31, 2016. Individual factors, prehospital factors, and hospital factors were extracted from the digital medical records of eligible cases. Data analysis was performed from March 1, 2020, to August 31, 2021.

**Exposures:**

Main exposure was onset-to-treatment time (time between symptom onset and aneurysm surgical treatment in hours) derived from medical records.

**Main Outcomes and Measures:**

Clinical characteristics, complications, and discharge destination were extracted from medical records and 12-month survival obtained from data linkage. The associations of onset-to-treatment time (in hours) with (1) discharge destination of survivors (home vs rehabilitation), (2) 12-month survival, and (3) neurologic complications (rebleed, delayed cerebral ischemia, meningitis, seizure, hydrocephalus, and delayed cerebral injury) were investigated using natural cubic splines in multivariable Cox proportional hazards and logistic regression models.

**Results:**

Of the 575 patients with aneurysmal SAH, 482 patients (mean [SD] age, 55.0 [14.5] years; 337 [69.9%] female) who received endovascular coiling or neurosurgical clipping were studied. A nonlinear association of treatment delay was found with the odds of being discharged home vs rehabilitation (effective *df* = 3.83 in the generalized additive model, χ^2^ test *P* = .002 for the 4-*df* cubic spline), with a similar nonlinear association remaining significant after adjustment for sex, treatment modality, severity, Charlson Comorbidity Index, history of hypertension, and hospital transfer (likelihood ratio test: *df* = 3, deviance = 9.57, χ^2^ test *P* = .02). Both unadjusted and adjusted cox regression models showed a nonlinear association between time to treatment and 12-month mortality with the lowest hazard of death with receipt of treatment at 12.5 hours after symptom onset, although the nonlinear term became nonsignificant upon adjustment. The odds of being discharged home were higher with treatment before 20 hours after onset, with the probability of being discharged home compared with rehabilitation or other hospital increased by approximately 10% when treatment was received within the first 12.5 hours after symptom onset and increased by an additional 5% from 12.5 to 20 hours. Time to treatment was not associated with any complications.

**Conclusions and Relevance:**

This cohort study found evidence that more favorable outcomes (discharge home and survival at 12 months) were achieved when surgical treatment occurred at approximately 12.5 hours. These findings provide more clarity around optimal timelines of treatment with people with aneurysmal SAH; however, additional studies are needed to confirm the findings.

## Key Points

**Question:**

What is the optimal time between symptom onset and treatment after aneurysmal subarachnoid hemorrhage (SAH) to maximize patient outcomes?

**Findings:**

In this cohort study, more favorable patient outcomes (discharge home and survival at 12 months) were observed when treatment occurred within 12.5 hours after aneurysmal SAH symptom onset. Treatment delay did not affect neurologic complications after aneurysmal SAH.

**Meaning:**

The findings of this study provide more information regarding the optimal timelines of surgical treatment for people with aSAH.

## Teaser

This cohort study of patients with aneurysmal subarachnoid hemorrhage assesses the associations between onset-to-treatment time and postsurgical outcomes.

## Introduction

Aneurysmal subarachnoid hemorrhage (SAH) is associated with significant morbidity and mortality.^1^ The optimal onset-to-treatment time for aneurysmal SAH remains poorly understood, with definitions of *early* and *delayed* treatment varying widely.^2^ Clinical guidelines from the American Heart Association and American Stroke Association in 2015 recommend treatment as early as feasible, whereas the 2013 European Stroke Organisation recommends intervention within 72 hours after onset of symptoms.

Neurosurgical or endovascular treatment within 24, 48, or 72 hours of onset has been associated with improved clinical outcomes.^2,3,4,5,6,7,8^ However, the data are heterogenous, with a significant risk of bias from the subjective stratification of timing, restriction to one treating center or treatment type, and exclusion of some groups of patients (eg, poor grade or transfers).^2,3,4,5,6,7,8^ Robust evidence on the optimal timing of surgery is therefore still lacking. In previous studies,^4,9^ the role of important factors, such as patient age, disease severity, and complications, on outcomes and the full range of aneurysmal SAH outcomes, such as survival, discharge destination, and complications, were not always considered. Robust evidence on the optimal timing of surgery is therefore still lacking. A better understanding of the optimal onset-to-treatment time after aneurysmal SAH will facilitate improved outcomes. This study examines the time delays in treatment of aneurysmal SAH and assesses the optimal treatment window to achieve the best outcomes after aneurysmal SAH treatment, including more frequent discharges home, increased 1-year survival after surgery, and fewer complications.

## Methods

The Reducing Delays in Aneurysmal Subarachnoid Hemorrhage (REDDISH) study is a retrospective, multicenter cohort study of all cases (N = 575) of aneurysmal SAH in patients admitted to 2 comprehensive cerebrovascular centers in Australia from January 1, 2010, to December 31, 2016. Data analysis was performed from March 1, 2020, to August 31, 2021. The study was approved by the Health and Medical Human Research Ethics Committees in Victoria and Tasmania with a waiver of informed consent for patients who died. All data were deidentified. Multiple overlapping data sources were used to identify potential cases of aneurysmal SAH (eAppendix 1 in the Supplement). Cases were confirmed based on neuroimaging findings and/or lumbar puncture results. This study followed the Strengthening the Reporting of Observational Studies in Epidemiology (STROBE) reporting guideline.

### Clinical Parameters

We used ambulance, emergency department, and radiologic records to extract demographic information, data on comorbidities, and clinical details. Onset-to-treatment time was defined as the time in hours between the onset of symptoms and the start of neurosurgical or endovascular treatment to secure the aneurysm. Where definite event date was missing, the first hospital arrival date was used as a proxy (n = 17). Where 24-hour time estimates for onset of symptoms were missing, the first ambulance call or 24-hour time at first hospital arrival was used as a proxy (n = 30) (eAppendix 1 in the Supplement).

### Outcomes

The primary outcome was discharge destination from the cerebrovascular centers (ie, home vs not) (including rehabilitation service, other hospital, or nursing home), which is used as a proxy of functional recovery and independence in survivors.^10^ Secondary outcomes included survival at 12 months (data linkage to National Death Index) and (2) complications of aneurysmal SAH using National Institute of Neurological Disorders and Stroke definitions (rebleed, posttreatment stroke, delayed cerebral ischemia, meningitis, seizure, hydrocephalus, and delayed cerebral injury).

### Exposure

Onset-to-treatment time was calculated as the difference in hours between the reported (or derived) date and time at onset of aneurysmal SAH symptoms (captured by triage records and ambulance notes) and the date and time at which neurosurgical or endovascular treatment was initiated (determined from procedure notes). A previous analysis^11^ within a subset of these cases for the state of Tasmania suggested that the prehospital time for most people with aneurysmal SAH is very short. To ensure potentially inaccurate patient-reported date and time of aneurysmal SAH symptom onset did not bias our results, we repeated all analyses excluding the prehospital time from onset-to-treatment time (eAppendix 2 in the Supplement).

### Covariates

Covariates that affect the prognosis of aneurysmal SAH and time to treatment^12^ were selected from the literature and clinical experience. Two indexes of severity based on first in-hospital assessments conducted before any procedures were performed included (1) the World Federation of Neurosurgical Societies (WFNS) score^13^ and the modified Fisher scale^14^ (eAppendix 1 in the Supplement provides definitions of score categories). Additional covariates included age, sex, history of hypertension, Charlson Comorbidity Index, treatment modality (neurosurgical or endovascular treatment), and hospital transfer (ie, direct admission vs transfer). There was no indication of nonlinear associations of age or severity indexes with any outcomes, so these variables were included as linear terms.

### Statistical Analysis

Among survivors, the continuous association of onset-to-treatment time with the odds of discharge to home compared with rehabilitation or other hospitals and the odds of developing complications (individually and combined) was assessed with multivariable logistic regression with a random effect to account for potential dependence arising in data collected from the same centers. We used log odds coefficients from adjusted models to derive partial predicted probability plots to show the chance of developing each outcome according to onset-to-treatment time. The association of onset-to-treatment time on survival at 12 months after aneurysmal SAH was investigated using multivariable Cox proportional hazards regression with a robust sandwich estimate of SEs to account for dependence within each of the 2 clinical centers.

In both the logistic and Cox proportional hazards regression models, we used natural cubic splines to explore the potential nonlinear, continuous association between onset to treatment and outcomes. Full details of the modeling approach, including model adjustments and interaction analyses with severity indexes and hospital transfer modality, are provided in eAppendix 3 in the Supplement. We performed a sensitivity analysis for each outcome to explore whether the results were consistent when limited to people treated within 72 hours and in those who received active treatment only (ie, excluding those treated patients who were given comfort measures only at some point after surgery). A 2-tailed *P* < .05 was considered statistically significant.

## Results

### Baseline Characteristics

**Figure 1.**
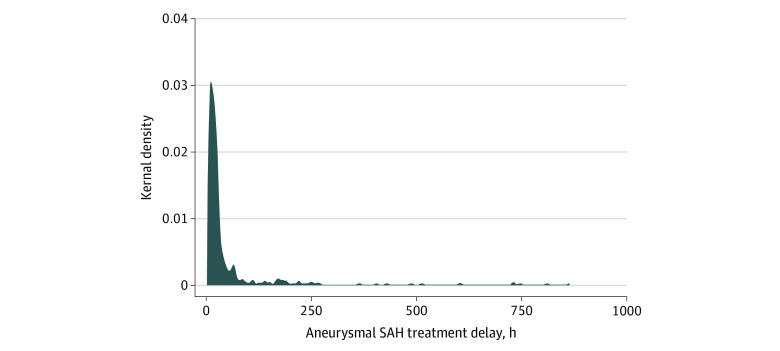
Distribution of Onset-to-Treatment Time for 482 Treated Participants in the Reducing Delays in Aneurysmal Subarachnoid Hemorrhage (SAH) Study Onset-to-treatment time is the time elapsed between symptoms onset and aneurysmal SAH treatment. A total of 295 participants had onset-to-treatment time delays of less than 24 hours, 99 had delays of 24 to 48 hours, and 80 had delays of greater than 48 hours (data were missing for 8 participants).

Of the 575 patients with aneurysmal SAH, 482 patients (mean [SD] age, 55.0 [14.5] years; 337 [69.9%] female) who were treated with neurosurgical or endovascular treatment were included in the study (Table 1; eResults and eTable 1 in the Supplement). Clinical characteristics of the 93 untreated patients (16.2%), including survival, time to death, and aneurysmal SAH severity, are presented in the eResults in the Supplement. These 93 patients were excluded from all analyses because the focus of the study was to link onset-to-treatment time to postsurgery prognosis. Time of onset was missing for 47 (9.7%) of the included patients; thus, first ambulance call (37 [7.6%]) or hospital arrival (10 [4.8%]) were used as proxies for time at symptom onset. Median onset-to-treatment time was 19.37 hours (range, 2 hours to 35 days) (Figure 1), and the distribution of symptom onset to treatment time was similar between sexes (eFigure 1 in the Supplement). Ventriculostomy and hematoma evacuation did not confound the associations between onset-to-treatment and any of the considered outcomes. Sensitivity analyses excluding those treated outside 72 hours (n = 53) and excluding those for whom active treatment was withdrawn (n = 64) showed results consistent with analyses that considered all patients.

**Table 1.** Characteristics of REDDISH Patients Included in the Analyses

Characteristic	Finding (N = 482)^a^
Age, mean (SD), y	55.0 (14.5)
Sex	
Female	337 (69.9)
Male	145 (30.1)
Hypertension	195 (40.4)
Smoking	
Missing	77 (16.8)
Current smoker	239 (49.6)
Ex-smoker	56 (11.6)
No smoking	110 (22.8)
CCI	
0	356 (73.8)
1-3	109 (22.6)
≥4	17 (3.5)
Modified Fisher scale score, median (IQR)	4 (1)
0	8 (1.6)
1	46 (9.5)
2	18 (3.7)
3	106 (21.9)
4	264 (54.7)
WFNS score, mean (SD)	2.28 (1.49)
1	235 (48.7)
2	92 (19.1)
3	22 (4.5)
4	44 (9.1)
5	81 (16.8)
Onset-to-treatment time, h	
Mean (SD)	49.5 (122.4)
Median (IQR)	19.5 (10.58-30.98)
Hospital transfer	254 (50.8)
Ventriculostomy	235 (48.7)
Hematoma evacuation	12 (3.9)
Treatment modality	
Clipping	186 (38.6)
Coiling	296 (61.4)

Abbreviations: CCI, Charlson Comorbidity Index; REDDISH; Reducing Delays in Aneurysmal Subarachnoid Hemorrhage study; WFNS, World Federation of Neurosurgical Societies.

^a^ Data are presented as number (percentage) of patients unless otherwise indicated.

### Discharge Destination

**Figure 2.**
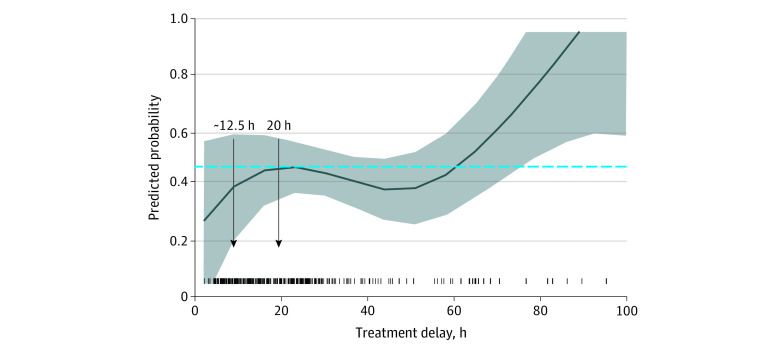
Association of Time to Treatment With the Odds of Being Discharged Home vs to a Rehabilitation Facility in Participants With Aneurysmal Subarachnoid Hemorrhage These partial predicted probability plots were derived from the adjusted logistic regression model with a 4-*df* natural cubic spline to model nonlinearity of effect. Shaded areas indicated 95% CIs.

In total, 80 patients (16%) died in hospital, 203 (42.8%) were discharged home, and 199 (41.3%) were discharged to rehabilitation services or other hospitals or nursing homes (mortality rate at 12 months was 17%). In the logistic regression model, there was a very strong nonlinear association of treatment delay with the odds of being discharged home vs rehabilitation (effective *df* = 3.83 in the generalized additive model, χ^2^ test *P* = .002 for the 4-*df* cubic spline), with a similar nonlinear association remaining significant after adjustment for sex, treatment modality, severity, Charlson Comorbidity Index, history of hypertension, and hospital transfer (likelihood ratio test: *df* = 3, deviance = 9.57, χ^2^ test *P* = .02). The odds of being discharged home were higher with treatment before 20 hours after onset, with the probability of being discharged home compared with rehabilitation or other hospital increased by approximately 10% when treatment was received within the first 12.5 hours after symptom onset and increased by an additional 5% from 12.5 to 20 hours. Beyond 20 hours, it decreased until approximately 60 hours after onset (Figure 2). The nonlinear association of time with treatment on the odds of being discharged home was not modified by any of the covariates, including WFNS score or treatment type (eTable 4 and eFigure 5 in the Supplement), and the shape of the nonlinear association was not different among those who received comfort care only measures (n = 64).

### 12-Month Survival

**Figure 3.**
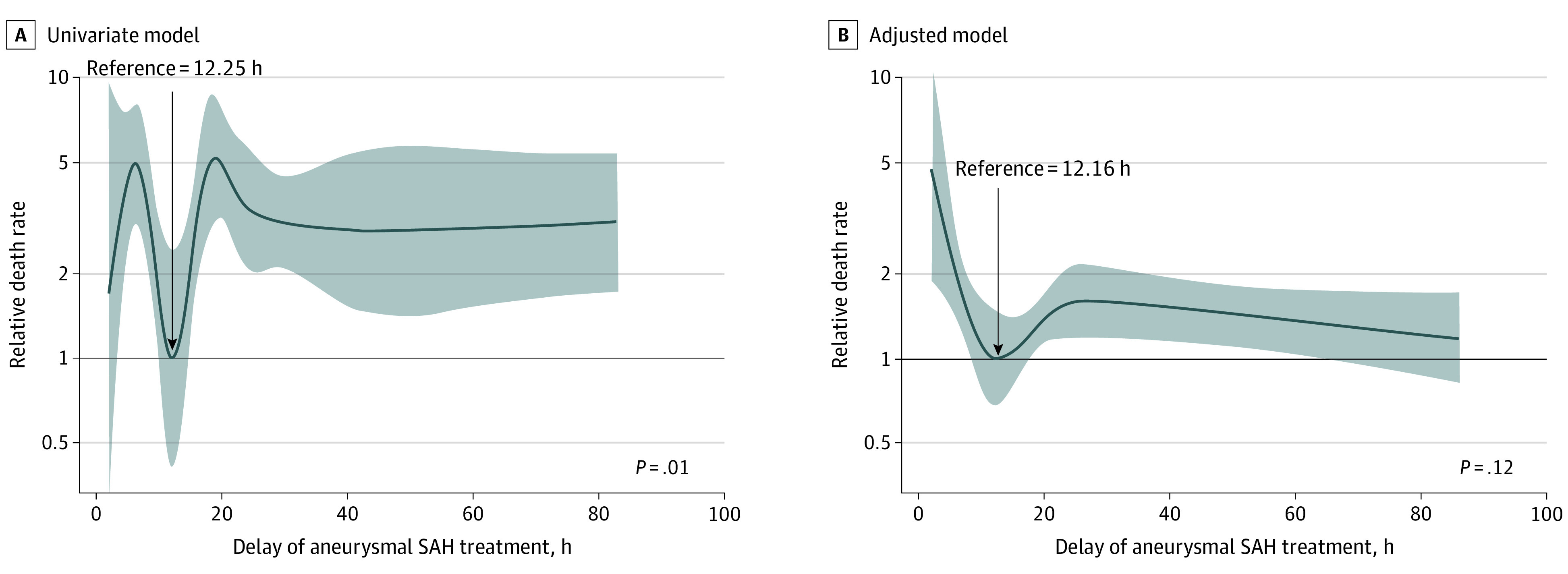
Relative Death Rates as a Function of Onset to Treatment Among 474 Participants Who Received Coiling or Clipping of Their Aneurysm Shaded areas indicate 95% CIs.

Overall, 400 of 482 treated patients (83.0%) were alive at 12 months. Among those who died, 67 deaths (81.7%) occurred with 30 days of symptom onset. Unadjusted survival models found a nonlinear association between the 12-month all-cause mortality hazard ratio (HR) on the delay in receiving aneurysmal SAH treatment (linear vs penalized cubic spline term for onset-to-treatment [*df* = 7] association in the univariable Cox proportional hazards regression models, χ^2^ = 14.39, *df* = 6, *P* = .01). The lowest hazard was estimated when receiving treatment at 12.25 hours after symptom onset. Using 12.25 hours as a reference (eg, risk = 1), we transformed the log HR curves to show the change in the relative risk of death at 12 months. There was evidence of decreasing hazard of death when treatment was received between 5 and 12.25 hours after onset, reaching its lowest point at 12.25 hours (risk = 1). The hazard of death then increased with treatment from 12.25 to 24 hours but still remained beneficial to survival, before plateauing at 4 times the risk, when treatment was received after 24 hours, compared with a reference time of 12.25 hours (Figure 3A).

On adjustment for covariates (sex, age, treatment modality, severity, comorbidities, and hospital transfer), the nonlinear association of onset-to-treatment time was attenuated. However, the shape of the association of treatment delay with the 12-month hazard of death from the adjusted model was similar to the shape of the unadjusted model with decreasing hazard of death with treatment from 0 to 12.5 hours, which was the treatment timing associated with the lowest hazard of death at 12 months (Figure 3). The hazard of death then increased from 12.16 hours to approximately 24 hours but was still associated with a benefit to survival that was diminished with treatment beyond 24 hours. Interaction terms between the nonlinear association of onset-to-treatment time and covariates, including severity of the aneurysmal SAH with WFNS score (log likelihood of −441.63; *P* = .18) or treatment type (log likelihood of −467.57; *P* = .25, were nonsignificant (eTable 2 in the Supplement). This finding shows that the shape of the association between time to treatment and survival did not differ significantly by these different patient groups (eFigures 2 and 3 in the Supplement).

### Aneurysmal SAH–Related Secondary Injuries

More than half of the treated patients experienced at least 1 of the 7 predefined complications after aneurysmal SAH (Table 2). Treatment delay was not associated with increased risk in any of the 7 individual secondary injuries or a combined metric of any complication (Table 2). Multinomial logistic regression models showed no evidence that treatment delay was associated with the total number of secondary injuries (eFigure 4 and eTable 3 in the Supplement).

**Table 2.** Prevalence of Aneurysm-Related Complications and Association Between Onset-to-Treatment Time and Secondary Injuries in Treated REDDISH Participants for Adjusted Models^a^

Complication	No. (%) of participants (N = 482)	Adjusted OR (95% CI)^a^	Nonlinearity of the risk over time^b^	Likelihood ratio test *P* value (linear vs cubic spline) for treatment delay^c^
Individual complications				
Stroke	100 (26.7)	1.00 (0.98-1.00)	1.00	.28
Rebleed	12 (2.5)	0.94 (0.89-1.01)	1.00	.26
Clinical deterioration	156 (32.3)	1.00 (0.99-1.00)	1.36	.48
Delayed cerebral ischemia	118 (24.4)	1.00 (0.98-1.00)	1.00	.88
Meningitis	57 (12.0)	0.99 (0.98-1.00)	1.00	.28
Seizure	40 (8.9)	0.99 (0.98-1.01)	3.04	.11
Hydrocephalus	301 (62.7)	1.00 (1.00-1.01)	3.26	.43
Any complication	254 (52.6)	1.00 (0.99-1.00)	1.20	.67
No. of complications			1.17	.42
0	221 (45.8)	NA	NA	NA
1-2	205 (45.5)	1.00 (1.00-1.01)	NA	NA
≥3	56 (11.6)	0.96 (0.97-1.00)	NA	NA

Abbreviations: NA, not applicable; OR, odds ratio; REDDISH: Reducing Delays in Aneurysmal Subarachnoid Hemorrhage study.

^a^ Adjustments include age, sex, procedure type, WFNS, modified Fisher scale score, Charlson Comorbidity Index, smoking, history of hypertension, ventriculostomy, and hematoma evacuation before treatment.

^b^ Nonlinearity assessed using effective *df* from univariate generalized additive model (see eAppendix 3 in the Supplement for details).

^c^ The nonlinear association between complications and treatment delay was assessed through likelihood ratio testing (*df* = 3, deviance = 9.57, χ^2^ test *P* = .02), comparing the univariate model with linear effect with the model with natural cubic spline term, where *df* was set to the rounded-down effective *df* value from the corresponding generalized additive model for treatment delay.

## Discussion

This cohort study is the first, to our knowledge, to characterize the nonlinear association between onset-to-treatment time for aneurysmal SAH against 12-month survival, complications, and discharge destination. Our analyses suggest that the optimal time window for receiving aneurysmal SAH treatment is up to approximately 12 hours postsymptom onset with decreasing evidence of benefit with treatment from 12 to 24 hours. Only 30% of the study participants with aneurysmal SAH received treatment within this time frame, highlighting the potential need to improve timely access to care for patients presenting to the hospital with aneurysmal SAH symptoms. Acknowledging the potential limitations of our study design, our findings provide guidance to improve clinical guidelines that currently recommend early treatment without a clear time target for clinicians.

The hazard of death at 12 months was lowest when treatment occurred up to 12.25 hours after aneurysmal SAH symptom onset but then increased from 12.25 hours onward before reaching a plateau from 24 hours onward. The association of onset-to-treatment time on 12-month survival was attenuated when adjusting for covariates but maintained the same shape as in the unadjusted data. The exception was the initial increase in hazard of death in the approximately 5 hours immediately after symptom onset that was not present in adjusted models or when people who died early (<48 hours after onset) were excluded. This finding reinforces that survival after aneurysmal SAH was associated with initial aneurysmal SAH severity. In support of the importance of onset-to-treatment time on outcome after aneurysmal SAH, the probability of being discharged home compared with a rehabilitation center or another hospital, as a proxy for functional outcomes, also increased significantly when treatment was received within the first 12.5 hours after symptom onset. Of interest was that the risk of developing common secondary injuries was not associated with onset-to-treatment time, with complications largely driven by the severity of the aneurysmal SAH. Our findings are supported by previous studies^2,3,4,5,6,7,8^ that used arbitrary categorical definitions of early treatment as opposed to our consideration of its continuous nature. These definitions range from ultra-early (eg, within 12 hours from onset) to 96 hours from onset. There is a tendency for shorter definitions of early in more recent publications,^2,3,4,5,6,7,8,15,16,17,18,19^ but these articles mostly focused on endovascular interventions only, excluding a large proportion of people with aneurysmal SAH treated neurosurgically. The analyses in our study are the first to consider the dynamic nature of onset-to-treatment time after aneurysmal SAH and a range of outcomes in a large, multicenter study.

The reasons for maximal survival benefit from treatment at approximately 12.5 hours after onset being beneficial for outcomes are unclear. In addition to the clear benefit of treatment in reducing rebleeding of the aneurysm, treatment time may be a surrogate for onset of intensive management to reduce intracranial pressure and improve cerebral perfusion, which may result in better neurologic recovery. Future research, ideally on prospective data, should be undertaken to better understand the components and mechanisms of how earlier treatment may aid recovery in aneurysmal SAH. Conducting randomized clinical trials of life-threatening conditions such as aneurysmal SAH is challenging. As an initial step, replication of our analyses in other studies with comparable measures of time to treatment, confounders, and outcomes would be warranted to confirm our findings. Advanced modeling techniques, such as inverse probability of treatment weighting to approximate randomization, could then be used in other studies if the time window identified in our study is confirmed in other cohorts.

This study suggests that medical treatment for aneurysmal SAH should ideally be provided within 12.5 hours after onset but not beyond 24 hours because these results were associated with improved 12-month survival and a greater likelihood of discharge home, independent of severity of the aneurysmal SAH and other confounders. Too few people with aneurysmal SAH are treated within the 12.5- or 24-hour window associated with the best outcomes. Interventions to reduce time to treatment may be warranted after confirmation of our findings in other cohorts.

The association between aneurysm severity, onset-to treatment time, and survival at 12 months is complex. Although the nonlinear effect of time-to-treatment on survival was the same among those with poor and good grade aneurysmal SAH, severity is associated with onset-to-treatment time and outcomes. We adjusted for a wide range of potentially important confounding factors associated with time to treatment and outcomes to increase the robustness of our findings. Nevertheless, it is difficult to tease apart the true association between time to treatment and survival at 12 months without introducing potential confounding by indication.

The debate on the timing of aneurysm treatment after aneurysmal SAH pivots on the balance of the temporal risk for fatal rebleeding vs the risk of intervention-related morbidity when intervening early on an acutely injured brain.^20^ The timing of intervention is controversial for patients with poor-grade aneurysmal SAH who are unconscious at hospital admission and potentially more prone to rebleeding because of unstable clot surrounding a ruptured aneurysm.^3,6,7^ Because of the high mortality and disability for poor-grade aneurysmal SAH, such patients are often treated conservatively. Our data suggest that early intervention (ideally within 12.5 hours but not beyond 24 hours) to secure the aneurysm together with medical treatment has the potential to increase discharge to home and survival, irrespective of severity and other patient characteristics.

Only 29% of people in this study received treatment with 12.5 hours, and only 60% received treatment within 24 hours, the latter being the currently accepted target for onset-to-treatment time.^7^ This finding is supported by a previous study^7^ in which only half of people with aneurysmal SAH were treated within 24 hours after onset and 15% to 60% patients were treated at 48 hours or later after onset of symptoms. Advocating for intervention within 12 hours potentially requires increased use of medical imaging facilities, rapid access to neurosurgical and neurointerventional expertise, potential out-of-hours treatment that necessitates increased staffing and on-call demands, and more robust and efficient patient workflow practices from initial paramedic contact to hospital treatment. Changes in practice related to timing of treatment have previously been reported in aneurysmal SAH. For example, recent evidence that it was unnecessary to delay angiography to avoid complications from advances in technique mean that, anecdotally, few neurosurgeons now delay angiography.^21^ Further research to establish factors associated with receiving treatment for aneurysmal SAH earlier and whether it is cost-effective are warranted to assist with implementation of time-to-treatment targets.

### Strengths and Limitations

This study has several strengths. Missing data were minimal (<2% overall). In contrast to previous studies,^2,3^ we had consecutive cases across 2 centers, including transferred cases, and all grades of aneurysmal SAH and treatment types, thereby facilitating a more complete picture of time to treatment and outcome. Nonetheless, future prospective studies to account for selection and measurement bias incorporating patient-reported outcome measures are warranted.

The current study also had several limitations. Its retrospective nature may result in bias in data capture and interpretation. However, we used rigorous case finding procedures and had minimal missing data, suggesting these factors are not significant sources of bias. The sample size may appear modest compared with some other aneurysmal SAH studies^2,3^ that used linked administrative data, but these studies do not have the necessary time-related variables for the type of analysis undertaken here. Routinely collected clinical records lack patient-reported outcome measures. Discharge destination was used as a surrogate marker of functional outcomes. This marker assumes those discharged home are more likely to be functionally independent, which is subject to some degree of error, depending on individual patients’ housing and caregiving support situation that could not be measured in the current study. To facilitate future research and evaluation, clinical services for people with aneurysmal SAH should consider embedding patient-reported outcome measures as routine. Because our aim was to identify the treatment window that maximized patients’ prognosis after surgery, excluding the 93 participants who were not treated will not result in survivor bias for this group. However, we cannot discount that given that 16% of the starting cohort was not treated (86% of whom subsequently died), there are likely to be approximately 20% of patients with SAH who die before they reach the hospital.^12^ Because the group of treated participants included in our analysis have a lower risk of death compared with the total group of people with aneurysmal SAH, a survivor bias may have resulted if we tried to generalize our findings to all those patients with aneurysmal SAH admitted to cerebrovascular centers.

## Conclusions

This cohort study suggests that treatment for aneurysmal SAH should ideally be provided within 12.5 hours after onset but not beyond 24 hours because this results in improved 12-month survival and a greater likelihood of discharge home, independent of severity of the aneurysmal SAH and other confounders. Too few people with aneurysmal SAH are treated within the 12.5- or 24-hour window associated with the best outcomes. Interventions to reduce time to treatment may be warranted if these findings are confirmed in other cohorts.

## Notes

Supplement. **eAppendix 1.** Supplemental Materials and Methods **eAppendix 2.** Sensitivity Analyses **eAppendix 3.** Supplemental Statistical Methods **eResults.** Supplement Results **eTable 1.** Characteristics of REDDISH Patients Who Received Surgical Clipping and Endovascular Clipping of Their SAH (N = 482) **eTable 2.** Analysis of Deviance Table of Nested Multivariable Cox Regression Models Investigating Modification of the Nonlinear Effect of Treatment Delay on All-Cause Mortality at 12 Months by SAH Severity and Treatment Modality **eTable 3.** Summary of Treatment Delay (in Hours) Stratified by Number of Medical Complications **eTable 4.** Analysis of Deviance Table of Nested Logistic Regression Models Investigating Modification of the Nonlinear Effect of Treatment Delay (Natural Cubic Spline (*df* = 4)) on the Odds of Being Discharged Home (vs Rehabilitation) by SAH Severity and Hospital Transfer Modality **eFigure 1.** Sex-Specific Kernel Density Plot Showing the Distribution of Treatment Delay (Time Elapsed Between Symptoms Onset and SAH Treatment (in Hours)) for the N = 482 Treated Reddish Participants (N_female_ = 337 (70%), N_male_ = 145 (30%) **eFigure 2.** Relative Death Rates as a Function of Delay in Receiving SAH Treatment (in Hours) Stratified by WFNS Scores Categories (WFNS ≥3 (Poor-Grade SAH (N = 125 (26.4%) vs WFNS <3 (Non–Poor Grade WFNS (N = 349 (73.6%)) Estimated From the Additive Cox Regression Model (Model 2) **eFigure 3.** Relative Death Rates as a Function of Delay in Receiving SAH Treatment (in Hours) Stratified by Treatment Modality (Coiling vs Clipping) (Derived From Model 5) **eFigure 4.** Simulated Predicted Probabilities of Having N0, 1-2 or 3 or More Medical Complications as a Function of Treatment Delay in Hours (2-100 Hours Post SAH Symptom Onset) Derived From the Adjusted Multivariate Multinomial Model With a Linear Effect of Treatment Delay **eFigure 5.** Forest Plot of Estimated ORs for the Adjusted Logistic Regression Model for Discharge Destination (Home vs Rehabilitation) With a Nonlinear Effect of Treatment Delay (Modeled as a Natural Cubic Spline of *df* = 4)
eReferences
