# Error in the Byline

**DOI:** 10.1001/jamanetworkopen.2022.2124

**Published:** 2022-02-17

In the Original Investigation titled “Association of Onset-to-Treatment Time With Discharge Destination, Mortality, and Complications Among Patients With Aneurysmal Subarachnoid Hemorrhage,”^1^ published January 21, 2022, the name of coauthor Julian Maingard, MBBS, was misspelled in the byline and in the article citation in the Supplement. This article has been corrected.^1^
