# Supplementary material for: Association of Onset-to-Treatment Time With Discharge Destination, Mortality, and Complications Among Patients With Aneurysmal Subarachnoid Hemorrhage
Source: JAMA Netw Open. 2022 Jan 21;5(1):e2144039. doi: 10.1001/jamanetworkopen.2021.44039 (PMC8783267; doi:10.1001/jamanetworkopen.2021.44039)
Supplement: Supplement. — eAppendix 1. Supplemental Materials and Methods eAppendix 2. Sensitivity Analyses eAppendix 3. Supplemental Statistical Methods eResults. Supplement Results eTable 1. Characteristics of REDDISH Patients Who Received Surgical Clipping and Endovascular Clipping of Their SAH (N = 482) eTable 2. Analysis of Deviance Table of Nested Multivariable Cox Regression Models Investigating Modification of the Nonlinear Effect of Treatment Delay on All-Cause Mortality at 12 Months by SAH Severity and Treatment Modality eTable 3. Summary of Treatment Delay (in Hours) Stratified by Number of Medical Complications eTable 4. Analysis of Deviance Table of Nested Logistic Regression Models Investigating Modification of the Nonlinear Effect of Treatment Delay (Natural Cubic Spline (df = 4)) on the Odds of Being Discharged Home (vs Rehabilitation) by SAH Severity and Hospital Transfer Modality eFigure 1. Sex-Specific Kernel Density Plot Showing the Distribution of Treatment Delay (Time Elapsed Between Symptoms Onset and SAH Treatment (in Hours)) for the N = 482 Treated Reddish Participants (Nfemale = 337 (70%), Nmale = 145 (30%) eFigure 2. Relative Death Rates as a Function of Delay in Receiving SAH Treatment (in Hours) Stratified by WFNS Scores Categories (WFNS ≥3 (Poor-Grade SAH (N = 125 (26.4%) vs WFNS <3 (Non–Poor Grade WFNS (N = 349 (73.6%)) Estimated From the Additive Cox Regression Model (Model 2) eFigure 3. Relative Death Rates as a Function of Delay in Receiving SAH Treatment (in Hours) Stratified by Treatment Modality (Coiling vs Clipping) (Derived From Model 5) eFigure 4. Simulated Predicted Probabilities of Having N0, 1-2 or 3 or More Medical Complications as a Function of Treatment Delay in Hours (2-100 Hours Post SAH Symptom Onset) Derived From the Adjusted Multivariate Multinomial Model With a Linear Effect of Treatment Delay eFigure 5. Forest Plot of Estimated ORs for the Adjusted Logistic Regression Model for Discharge Destination (Home vs Rehabilitation) With a Nonlinea [file jamanetwopen-e2144039-s001.pdf]

Supplementary Online Content

Buscot MJ, Chandra RV, Mainguard J, et al. Association of onset-to-treatment time with discharge destination, mortality, and complications among patients with aneurysmal subarachnoid hemorrhage. *JAMA Netw Open*. 2022;5(1):e2144039. doi:10.1001/jamanetworkopen.2021.44039

eAppendix 1. Supplemental Materials and Methods

eAppendix 2. Sensitivity Analyses

eAppendix 3. Supplemental Statistical Methods

eResults. Supplement Results

eTable 1. Characteristics of REDDISH Patients Who Received Surgical Clipping and Endovascular Clipping of Their aSAH (N=482)

eTable 2. Analysis of Deviance Table of Nested Multivariable Cox Regression Models Investigating Modification of the Nonlinear Effect of Treatment Delay on All-Cause Mortality at 12 Months by aSAH Severity and Treatment Modality

eTable 3. Summary of Treatment Delay (in Hours) Stratified by Number of Medical Complications

eTable 4. Analysis of Deviance Table of Nested Logistic Regression Models Investigating Modification of the Nonlinear Effect of Treatment Delay (Natural Cubic Spline (*df* =4)) on the Odds of Being Discharged Home (vs Rehabilitation) by aSAH Severity and Hospital Transfer Modality

eFigure 1. Sex-Specific Kernel Density Plot Showing the Distribution of Treatment Delay (Time Elapsed Between Symptoms Onset and aSAH Treatment (in Hours)) for the N=482 Treated Reddish Participants (N_{female}=337 (70%), N_{male}=145 (30%)

eFigure 2. Relative Death Rates as a Function of Delay in Receiving aSAH Treatment (in Hours) Stratified by WFNS Scores Categories (WFNS ≥3 (Poor-Grade aSAH (N=125 (26.4%) vs WFNS< 3 (Non-Poor Grade WFNS (N=349 (73.6%)) Estimated From the Additive Cox Regression Model (Model 2)

eFigure 3. Relative Death Rates as a Function of Delay in Receiving aSAH Treatment (in Hours) Stratified by Treatment Modality (Coiling vs Clipping) (Derived From Model 5)

eFigure 4. Simulated Predicted Probabilities of Having No, 1-2 or 3 or More Medical Complications as a Function of Treatment Delay in Hours (2 to 100 Hours Post aSAH Symptom Onset) Derived From the Adjusted Multivariate Multinomial Model With a Linear Effect of Treatment Delay

eFigure 5. Forest Plot of Estimated ORs for the Adjusted Logistic Regression Model for Discharge Destination (Home vs Rehabilitation) With a Nonlinear Effect of Treatment Delay (Modelled as a Natural Cubic Spline of *df* =4)

eReferences

This supplementary material has been provided by the authors to give readers additional information about their work.

© 2022 Buscot MJ et al. *JAMA Network Open.*

eAppendix 1. Supplemental Methods

As reported elsewhere,[31] multiple overlapping sources were used to identify the cases including admission, discharge and ward lists for emergency, neurosurgical and radiology departments across the tertiary centres and referring hospitals. A combination of International Classification of Diseases 10 codes (160.0-160.9, 167.1 and 169.0), as either a primary or secondary diagnosis, and keyword searches were used to ascertain potential cases. A standardised abstraction form using data from radiology, pathology and surgical reports, as well as discharge letters were used to confirm first ever aSAH. Potential cases were coded by one researcher in each site, and a neurosurgeon and a neuro-interventional radiologist resolved any discrepancy in diagnosis. For those who received the two types of procedures (N=4), 'onset-to-treatment'time was defined as the time elapsed between symptoms onset and the first procedure.

Clinical parameters

We used ambulance, emergency, and radiology records to extract demographic information, baseline comorbidities, and clinical details including aSAH complications, severity indices (World Federation of Neurological Surgeons (WFNS)[14] and modified Fisher grade[15]), and treatment type (endovascular clipping (EVT) or neurosurgical clipping (NST)). Complications during hospitalisation were extracted from medical records while 12-month survival post-aSAH was determined using data linkage with the Australian Institute of Health and Welfare.

Definitions of WFNS and modified Fisher scale

WFNS grades ranged from 1 to 5 (grade 1: GCS 15, no motor deficit.; grade 2: GCS 13-14 without deficit; grade 3: GCS 13-14 with focal neurological deficit; grade 4: GCS 7-12, with or without deficit.; grade 5: GC <7 , with or without deficit), and the modified Fisher scale ranged from 0 to 4 (grade 0– no SAH or IVH, grade I – no blood, grade II – diffuse deposition of SAH without clots or layers of blood >1mm, grade III – localized clots and/or vertical layers of blood 1mm or > thickness, grade IV – diffuse or no subarachnoid blood but intracerebral or intraventricular clots)[1,2].

The modified Fisher scale indicates the risk of developing vasospasm (it progressively increases with each grade**).** The classification is as follows[3]:

- **grade 0**
  -
  - no subarachnoid haemorrhage (SAH)
  - no intraventricular haemorrhage (IVH)
  - incidence of symptomatic vasospasm: 0% [3]
- **grade 1**
  - focal or diffuse, thin SAH
  - no IVH
  - the incidence of symptomatic vasospasm: 24%
- **grade 2**
  - thin focal or diffuse SAH
  - IVH present

© 2022 Buscot MJ et al. *JAMA Network Open.*

    - the incidence of symptomatic vasospasm: 33%
- **grade 3**
    - thick focal or diffuse SAH
    - no IVH
    - the incidence of symptomatic vasospasm: 33%
- **grade 4**
    - thick focal or diffuse SAH
    - IVH present
    - the incidence of symptomatic vasospasm: 40%

For the purpose of model adjustment in the present study, WFNS scores were categorised as good (WFNS 1-2) or poor (WFNS≥3) using existing definitions [14]. Since very few patients had grade 0 and grade 1 Fisher scale (i.e. 8 (<1%) and 18 (3.4%), respectively), the modified Fisher scale was categorised (≤2, 3, 4, lower scores indicating better prognosis. Exploratory single predictor models did not suggest any non-linear effect of these two indices of severity on any of the considered outcomes

© 2022 Buscot MJ et al. *JAMA Network Open.*

eAppendix 2. Sensitivity Analyses

Multiple overlapping sources of information were used to provide robust information on key exposures, covariates and outcomes for this cohort. There was very minimal missing data, and while time and date stamps for ambulance phone calls, hospital arrivals and procedures times are likely to be highly accurate, the potentially least accurate time may be the patient reported date and time of aSAH symptom onset, which was captured from triage records but also ambulance notes. Our previous analyses within a subset of these cases for the state of Tasmania suggests that the pre-hospital time for most people with aSAH is very short.[1] Therefore, even if there is error in the pre-hospital time, this is unlikely to have affected our results greatly. To examine this, we have repeated our analyses on the time to treatment excluding the pre-hospital time. For these sensitivity analyses, the relevant time variable was defined as 'time- to- first hospitla arrival', defined as the time between the first hospital arrival and the time at which treatment was received (in hours). The distribution of this variable was very similar to the 'onset-to-treatment time' (Fig 1.), with the exception of less ' extreme' observations in the right tail. Bland-Altman plot (Fig 2.), and a Pearson's product moment correlation of 0.74 also suggest very high agreement between these two time measures.

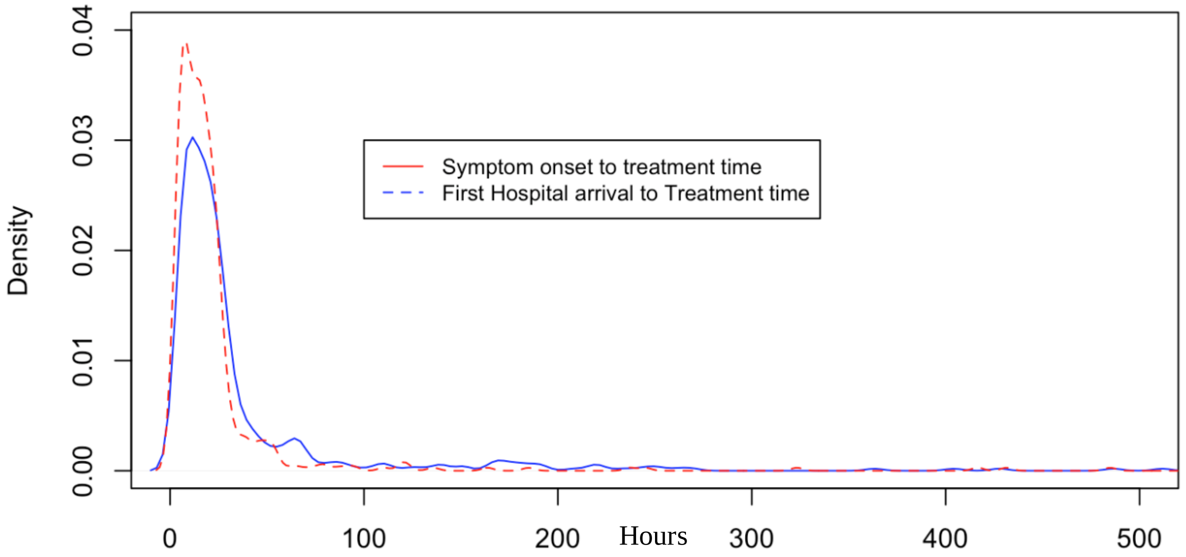

Kernel density plot of the time variable used in the main analyses ('Symptom onset to treatment time') and the time variable used in the sensitivity analyses ('First hospital arrival to treatment time).

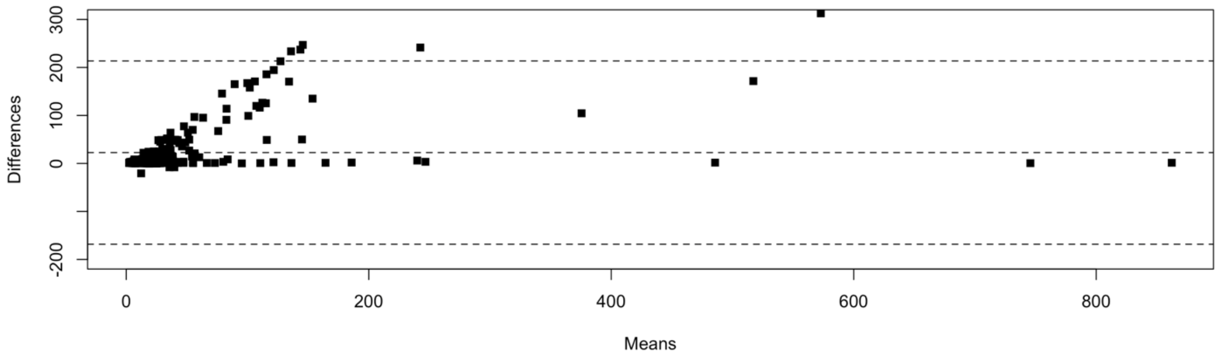

Bland-Altman plot of the differences between 'Symptom onset-to-treatment' time (i.e. original time variable) and 'First hospital arrival-to-treatment'time (i.e. time variable used for sensitivity analyses)

© 2022 Buscot MJ et al. *JAMA Network Open.*

Results of sensitivity analyses conducted with ‘First Hospital arrival to treatment’ time as an alternate exposure for the ‘time delay’ variable showed essentially the same patterns with all 3 outcomes, suggesting ~12.5 hours post-hospital arrival as the optimal window for aSAH intervention in terms of reducing the odds of being discharged to rehabilitation and maximizing survival at 1 year, with slightly different absolute values due to the shortened overall time to treatment by excluding pre-hospital times. Survival analyses still suggest ~12.3 hours as the treatment time that maximises survival at one year (i.e. similar to the original analysis the non-linear cubic term is significant in the univariate model (p-val =0.02) but not for the adjusted model (p-val=0.09)) (Fig 3.).

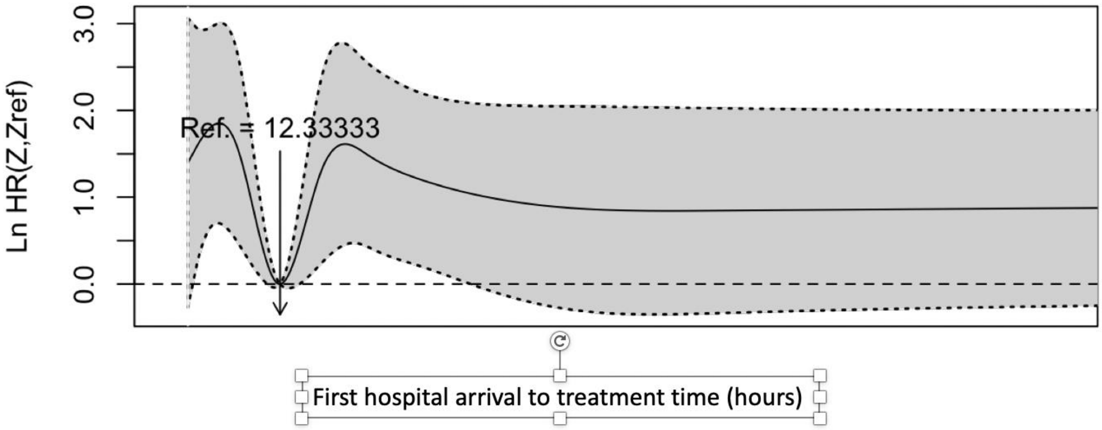

We still were unable to detect an association between this new time variable an any of the considered post-stroke complications. However, the relationship between the odds of being discharged home vs. being discharged to a rehabilitation service and this ‘First hospital arrival to treatment’ time follows the same shape as the one observed with 'time from onset to treatment', and the non-linear effect is still highly significant even in adjusted models (Fig 4.), suggesting that the odds of being discharged to rehabilitation decrease steeply within the initial 12.5 hours and are the lowers at ~20 hours between time of first hospital presentation and treatment of the aneurysm.

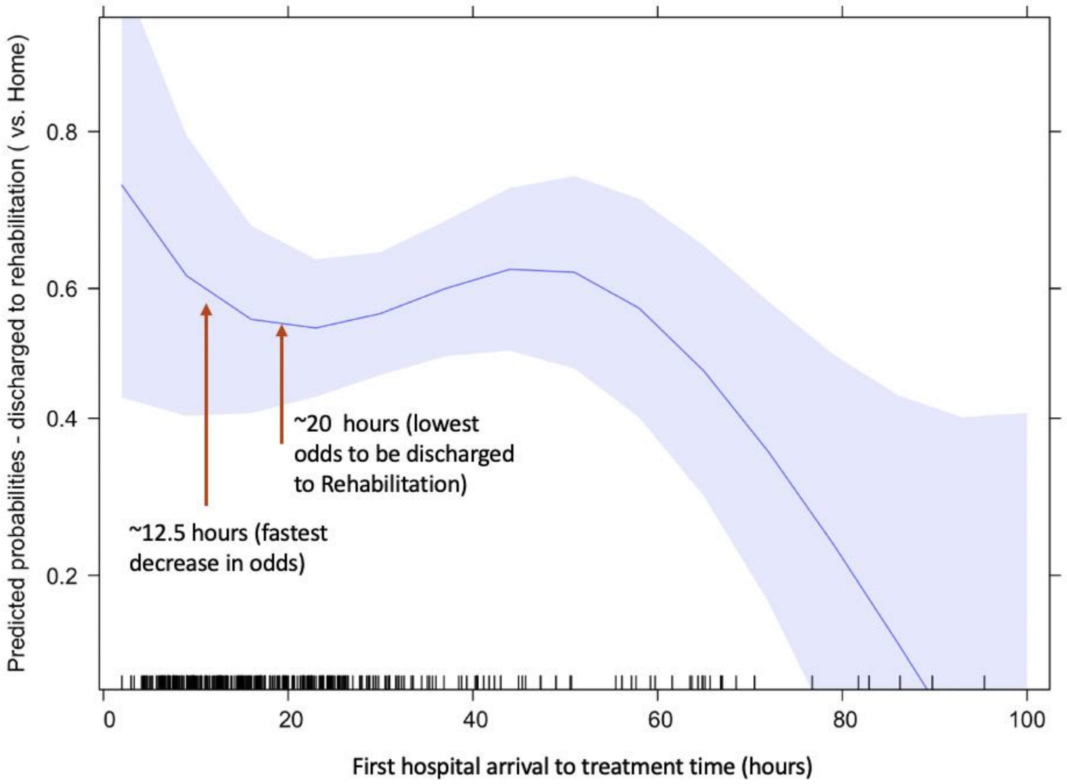

© 2022 Buscot MJ et al. *JAMA Network Open.*

eAppendix 3. Supplemental Statistical Methods

Non-linear effects of 'onset-to-treatment' time and other covariates: Natural cubic splines
For survival models, the optimal degrees of freedom for non-parametric smoothing terms in multivariable Cox models with nonlinear covariate effects was chosen to minimize the corrected Akaike's Information Criterion (AICc) (Meira-Machado et al. 2013). For logistic regression models (i.e. discharge destination and secondary complications models), the non-linearity of 'treatment delay' was assessed using the estimated degrees of freedom (edf) derived from exploratory univariate generalized additive models (GAM). The edf is a summary statistic of GAM models that reflects the degree of non-linearity of a curve (Wood 2006) (i.e. an edf equal to 1 is equivalent to a linear relationship, $1 < edf \leq 2$ is considered a weakly non-linear relationship, and $edf > 2$ implies a highly non-linear relationship). For complications where GAMs with edf> 2, natural cubic splines of degree of freedom equal to rounded-up edf values were introduced in logistic models, to model non-linear effect of time on the probability of developing complications.

Using the approaches highlighted in the paragraph above, in addition to the potential non-linear effect of 'onset-to-treatment time' on each outcome, we investigated the non-linear effect of other chosen continuous covariates (i.e. age, WFNS, and fisher scale) in single-predictors models. There was no indication of non-linear effect of age, WFNS and Fisher score on survival, discharge destination or complications. Because very few people had modified Fisher scale of grade 0 and 1, we decided to collapse them into 3 categories (≤2, 3, and 4) in adjusted models. In addition, for the purpose of model adjustment, since the effect of WFNS was not non-linear, we chose to classify WFNS as good grade (WFNS= 1-2) vs. poor grade (WFNS>3) using existing definitions[2].

Significance of non-linear terms and extraction of 'optimal' onset-to-treatment time from non-linear models

For all models, the significance of the non-linear effects of time on the probability of being discharged home and of developing each complication, and on the Hazard Ratio of death at 12 months was assessed through sequential likelihood ratio testing (LRT tests, where the null hypothesis was that the relationship was linear).

Where the non-linear effects were significant, local extrema in the time-dependant log hazard ratio curves (i.e. survival models) and predicted probability curves (i.e. logistic. Regression models) were extracted using the first derivative method. These points represent the time of greatest survival, greatest discharge home and lowest odds of developing each secondary injury. For 12 month survival, the point in time that was associated with lowest hazard of death was used to derive relative death rate as a function of time-to-treatment, representing the risk of death at 12 month overtime relative to the time where the survival was maximised.

Covariate adjustment
Models were adjusted for age, gender, procedure type, severity, direct admission vs. transfer, ventriculostomy, haematoma evacuation and co-morbidities. To determine if these covariates modified the effect of 'onset-to-treatment' on outcomes we used product terms between the non-linear time to treatment (specified as a natural cubic splines) and covariates. A significant ($p \leq 0.05$) interaction term indicates that the effect of time-to-treatment on a given outcome was different according to levels of that covariate.

© 2022 Buscot MJ et al. *JAMA Network Open.*

eResults. Supplementary Results

69.2% treated participants were female and median age at event was 55.5 (SD=14.5) years, and females were on average 4 years older than male patients. Amongst the 482 treated cases, more patients underwent treatment with endovascular coiling than surgical clipping (61.4% versus 38.6%, respectively, (**Table 1**)). Patients who received clipping were on average 4 years younger than those who received endovascular coiling (**Table 1**). Time delay in hours between the estimated time at symptom onset and the time at treatment was available for N=474 (98.3%) participants.

Median treatment delay in men was longer by ~3 hours on average than female, but the difference was non-significant (**Table 2**).67 patients (13.9%) and 82 (17%) treated patients died within 1-month and 1-year post event, respectively, with no sex-differences (**Supplementary Table II**). The proportion of patients who died of all-cause mortality at 1 month and 1-year post-event were not significantly different between the two treatment modalities (**Supplementary Table I**). There was no difference in aSAH severity indices between treatment procedure groups or between genders (**Supplementary Table I and Supplementary Table II**). N=125 operated patients (26.4%) were graded as poor-grade aSAH upon admission (WFNS scores ≥ 3).

Amongst our 575 confirmed cases, 93 were not treated for aSAH and therefore excluded from all our analyses.
A total of 161 reddish participants died within a year post-symptom onset.
*7 people (out of 575) died within the same day as symptom onset: all of these 7 patients were untreated
* 39 patients (out of 575) died the next day (between 24 and 48 hours post-onset): 36 of these received no treatment and only 3 had clipping or coiling

Treatment modality	N (%)	Died (n(%))	Survived (n(%)
Not treated	93 (32.3)	79 (86.0)	14 (14.0)
Clipping	186 (51.5)	32 (17.2)	154 (82.8)
Coiling	293(16.2)	50 (16.8)	246 (83.2)
Total	575	161(28)	414 (72)

The 93 untreated participants also experience earlier deaths, with average 'onset- to death' time of 3.3 (sd=4.1) days compared to 21 (sd=26.5) day in the treated group (see kernel density plot below)

Kernel density plot of 'onset to death' time in the N=79 untreated patients who died (black line) and the N=82 treated patients who died (Blue line).

© 2022 Buscot MJ et al. *JAMA Network Open.*

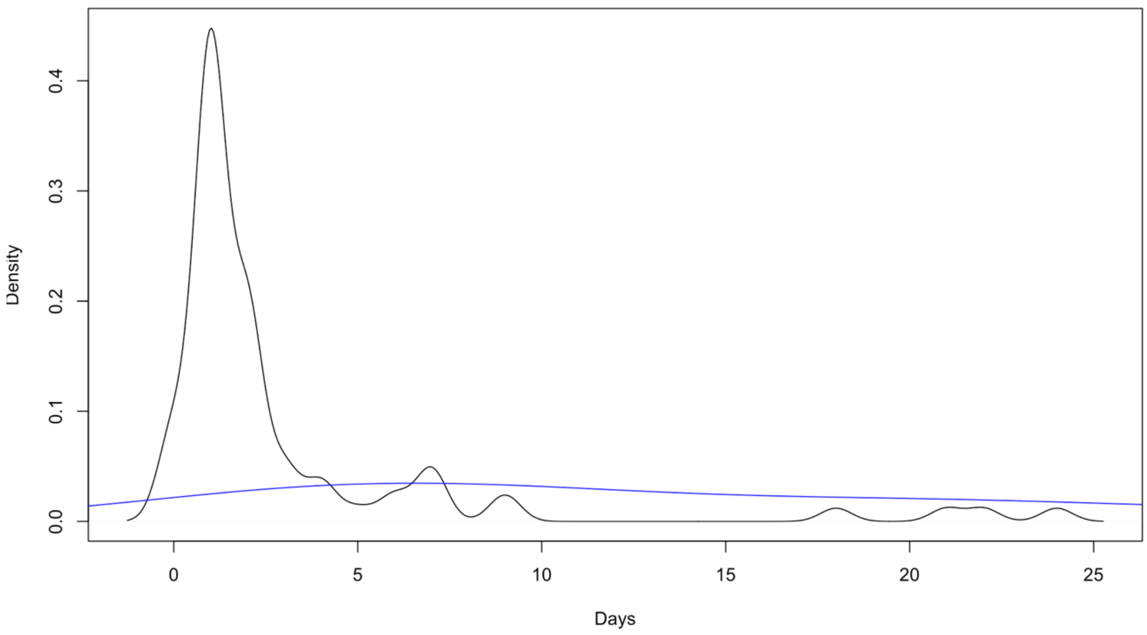

The following table presents characteristics of the N=93 untreated participants:

Untreated patients were older than the 482 treated patients on average, had a more frequent history of hypertension, and their aSAH was more severe based on fisher scale and WFNS.

**Characteristic**	**N (%)***
**Age** (in years), mean (SD)	66.2 (14.5)
**$N_{Females}$ (%)**	61 (65.5)
**Hypertension**	53 (56.9)
**Charlson comorbidity index (CCI)**	
CCI score =0	59 (63.4)
CCI score =1 to 3	32 (34.4)
CCI score ≥4	1 (1)
**Modified Fisher scale,** median (IQR)	5 (1)
3 and less	4 (4.3)
4	5 (5.4)
5	66 (70.9)
**WFNS** (mean(SD))	3.9 (1.49)
Less than 3	25 (26.8)
3 or more	64 (68.8)

© 2022 Buscot MJ et al. *JAMA Network Open.*

eTable 1. Characteristics of REDDISH Patients Who Received Surgical Clipping and Endovascular Clipping of Their aSAH (N=482)

	**Surgical clipping**	**Endovascular coiling**	**P-value**[a]
**N (%)**	186 (38.6)	296 (61.4)	**<0.01***
**$N_{Females}$ (%)**	126 (67.7)	209 (70.6%)	0.60
**Age** (in years)(mean (sd)	52.39 (14.6)	56.7 (14.3)	**<0.01***
**Delay treatment** (in hours)(mean (sd)	58.3(151.3)/19.7	43.8(99.5)/19.2	0.2487
/median)			
**Modified Fisher scale** (mean(sd))	4.31(1.09)	4.25 (1.01)	0.54
Grade 0	1 (0.6)	7 (2.4)	
Grade 1	18 (11)	28 (9.9)	
Grade 2	7 (4.3)	11 (3.9)	
Grade 3	48 (29.8)	58 (20.6)	
Grade 4	87 (54.1)	177 62.9)(	
**WFNS** (mean(sd))	2.28 (1.49)	2.26 (1.64)	0.39
Grade 1	92 (50.5)	143 (48.9)	
Grade 2	30 (16.5)	62 (21.2)	
Grade 3	12 (6.6)	10 (3.4)	
Grade 4	19 (10.4)	25 (8.5)	
Grade 5	29 (15.9)	52 (17.8)	
**N_{deaths} (%) -1 month**	26 (13.9)	41 (13.8)	0.94
**N_{deaths} (%) -12 months**	32 (17.2)	50 (17.5)	0.85

[a]:p-values for Student t-tests or chis-square tests for difference in means and proportions, respectively.

© 2022 Buscot MJ et al. *JAMA Network Open.*

eTable 2. Analysis of Deviance Table of Nested Multivariable Cox Regression Models Investigating Modification of the Nonlinear Effect of Treatment Delay on All-Cause Mortality at 12 Months by aSAH Severity and Treatment Modality

**Effect modification**		**Log lik**	**Df**	**P(>|Chi|)**	**AIC**
**WFNS category**	**Model 1 –** Univariate	-472.16	7		958.31
	$Surv_{12\ months}$ ~ cubic.spline				
	(treatment delay)				
	**Model 2 -** Additive	-446.71	8	**<0.01**	909.20
	$Surv_{12\ months}$ ~ cubic.spline				
	(treatment delay) +				
	$WFNS_{category}$				
	**Model 3 -** Interaction	-441.63	15	0.18	913.26
	$Surv_{12\ months}$ ~ cubic.spline (treatment delay) × $WFNS_{category}$				
	$WFNS_{category}$				
**Treatment modality**	**Model 4 -** Univariate	-472.16	7		958.31
	$Surv_{12\ months}$ ~ cubic.spline				
	(treatment delay)				
	**Model 5 -** Additive	-472.07	8	0.68	960.2
	$Surv_{12\ months}$ ~ cubic.spline				
	(treatment delay) +				
	$Treatment_{type}$				
	**Model 6 -** Interaction	-467.57	15	0.25	965.7
	$Surv_{12\ months}$ ~ cubic.spline (treatment delay) × $Treatment_{type}$				

© 2022 Buscot MJ et al. *JAMA Network Open.*

eTable 3. Summary of Treatment Delay (in Hours) Stratified by Number of Medical Complications

	**Treatment delays summaries (in hours)**		
	**Mean (sd)**	**Median**	**range**
**No complications**	51.4(143.3)	19.6	2-1555
**1-2 complications**	54.9 (112.9)	20.0	3-809
**3-6 complications**	21.9 (19.3)	16.01	3.33-110.96

© 2022 Buscot MJ et al. *JAMA Network Open.*

eTable 4. Analysis of Deviance Table of Nested Logistic Regression Models Investigating Modification of the Nonlinear Effect of Treatment Delay (Natural Cubic Spline (*df* =4)) on the Odds of Being Discharged Home (vs Rehabilitation) by aSAH Severity and Hospital Transfer Modality

**Effect modification**		**Log likelihood**	**Df**	**P(>|Chi|)**	**AIC**
**WFNS**	**Model 1 –** univariate	-238.5	5		486.7
**Category**	Discharge destination ~ cubic.spline				
	(treatment delay)				
	**Model 2 –** Additive	-186.62	10	**<0.01**	393.25
	Discharge destination ~ cubic.spline				
	(treatment delay) + $WFNS_{category}$ +				
	$Transfer_{type}$+gender+procedure_type+				
	FISHER $SCALE_{category}$				
	**Model 3 –** Interaction	-185.1	14	0.28	399.05
	Discharge destination ~ cubic.spline				
	(treatment delay) *$WFNS_{category}$ +				
	$Transfer_{type}$+gender+procedure_type+				
	FISHER $SCALE_{category}$				
**Hospital**	**Model 4 –** univariate	-238.5	5		486.7
**Transfer**	Discharge destination ~ cubic.spline				
**Modality**	(treatment delay)				
	**Model 5 –** Additive	-186.62	10	**<0.01**	393.25
	Discharge destination ~ cubic.spline				
	(treatment delay) + $WFNS_{category}$ +				
	$Transfer_{type}$+gender+procedure_type+				
	FISHER $SCALE_{category}$				
	**Model 6 -**Interaction	-186.04	14	0.69	410.1
	Discharge destination ~ cubic.spline				
	(treatment delay)* $Transfer_{type}$				
	+$WFNS_{category}$ +gender+procedure_type+ FISHER $SCALE_{category}$				

© 2022 Buscot MJ et al. *JAMA Network Open.*

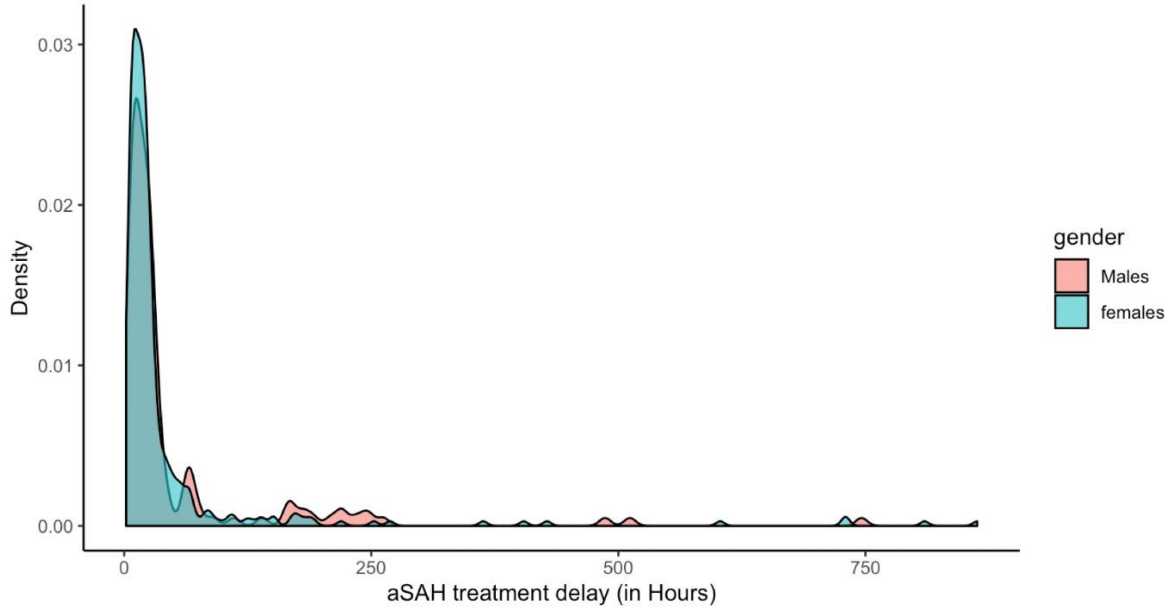

eFigure 1. Sex-Specific Kernel Density Plot Showing the Distribution of Treatment Delay (Time Elapsed Between Symptoms Onset and aSAH Treatment (in Hours)) for the N=482 Treated Reddish Participants (N_{female}=337 (70%), N_{male}=145 (30%)

© 2022 Buscot MJ et al. *JAMA Network Open.*

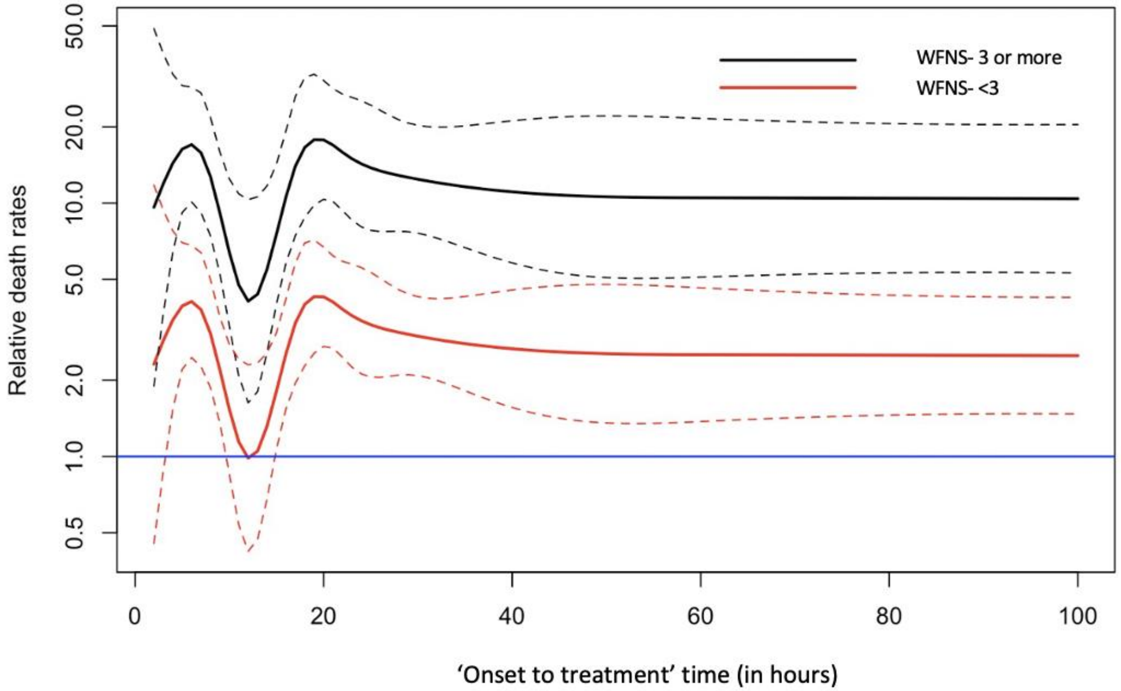

eFigure 2. Relative Death Rates as a Function of Delay in Receiving aSAH Treatment (in Hours) Stratified by WFNS Scores Categories (WFNS ≥3 (Poor-Grade aSAH (N=125 (26.4%) vs WFNS< 3 (Non-Poor Grade WFNS (N=349 (73.6%)) Estimated From the Additive Cox Regression Model (Model 2)

The fixed reference (12.25 hours (continuous blue line), was chosen as the "onset to treatment' time where the log Hazard ratio curve was minimized for both groups. (i.e. the risk of death at 12 months = 1 when treatment was received at 12.25 hours post symptom onset**).** Nonparametric estimates of the dependence of all cause death mortality at 12 months on the delay in receiving aSAH treatment in hours were restricted to the interval between 0 and 100 hours post aSAH symptom onset for each group. In model 2, HR for WFNS≥3 (vs. WFNS< 3) is 4.16 (95%CI: 2.61-6.52, p-val<0.01).

© 2022 Buscot MJ et al. *JAMA Network Open.*

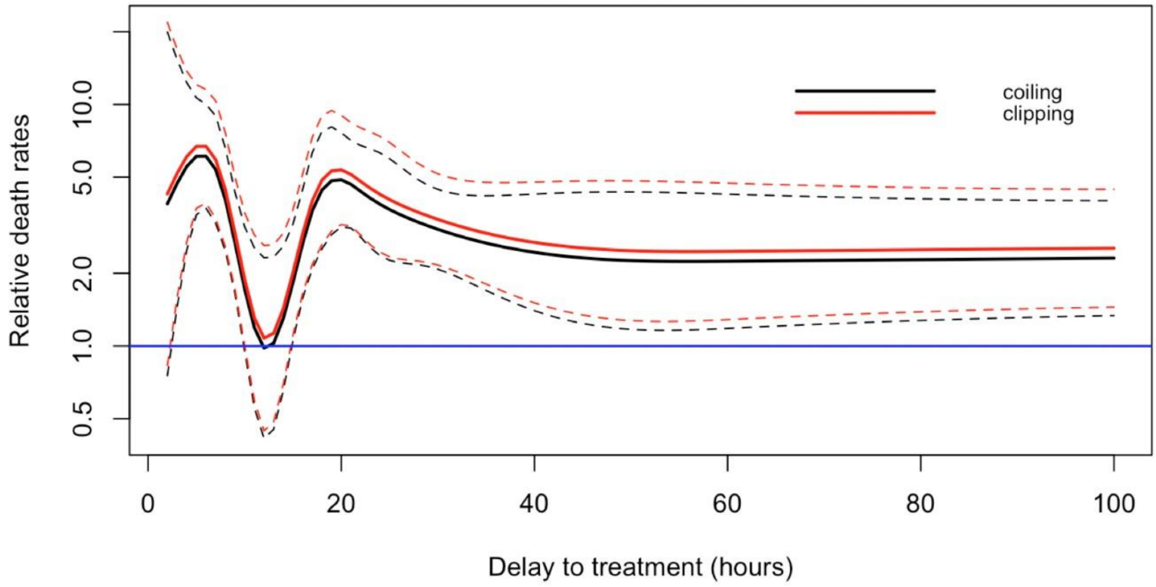

eFigure 3. Relative Death Rates as a Function of Delay in Receiving aSAH Treatment (in Hours) Stratified by Treatment Modality (Coiling vs Clipping) (Derived From Model 5)

The fixed reference (12.25 hours (continuous blue line), was chosen as the "time to treatment' where the log Hazard ratio curve was minimized for both groups. (i.e. the risk of death at 12 months = 1 when treatment was received at 12.25 hours post symptom onset). Nonparametric estimates of the dependence of all cause death mortality at 12 months on the delay in receiving aSAH treatment in hours were restricted to the interval between 0 and 100 hours post aSAH symptom onset for each group.

© 2022 Buscot MJ et al. *JAMA Network Open.*

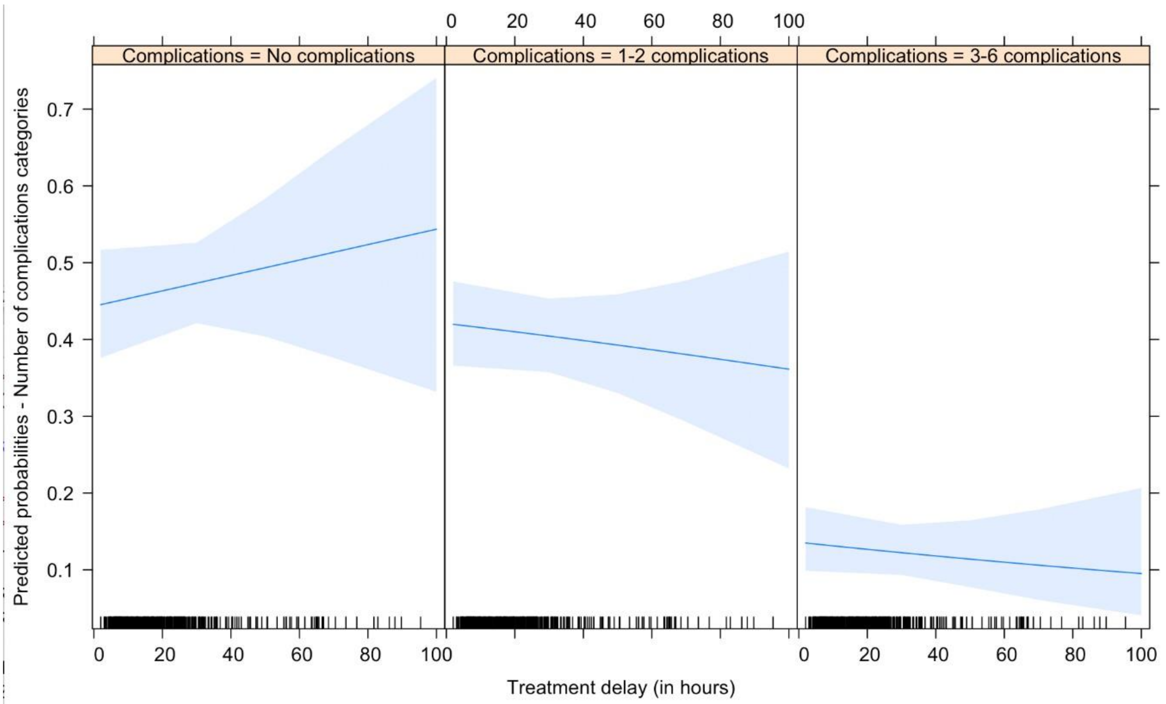

eFigure 4. Simulated Predicted Probabilities of Having No, 1-2 or 3 or More Medical Complications as a Function of Treatment Delay in Hours (2 to 100 Hours Post aSAH Symptom Onset) Derived From the Adjusted Multivariate Multinomial Model With a Linear Effect of Treatment Delay

(Note: covariates were fixed for prediction purposes, so that probabilities shown are for a >55 years old female patient who received endovascular coiling and had a WFNS score <4, a modified fisher scale <3 at hospital admission)

© 2022 Buscot MJ et al. *JAMA Network Open.*

eFigure 5. Forest Plot of Estimated ORs for the Adjusted Logistic Regression Model for Discharge Destination (Home vs Rehabilitation) With a Nonlinear Effect of Treatment Delay (Modelled as a Natural Cubic Spline of *df* =4)

Variable		N	Odds ratio	p
**ns(DelayTreatment, 4)**		1320		
**transfer**		330	1.02 (0.61, 1.72)	0.94
**gender**	Males	98	Reference	
	females	232	1.41 (0.82, 2.44)	0.22
**procedure_type2**	clipping	115	Reference	
	coiling	215	0.72 (0.42, 1.21)	0.21
**wfns.categories**	WFNS <4	257	Reference	
	WFNS- 4 or more	73	9.14 (4.44, 20.88)	<0.001
**fisher_scale.categories**	3 and less	136	Reference	
	4	194	2.58 (1.55, 4.34)	<0.001
**(Intercept)**			0.69 (0.13, 3.65)	0.66
**ns(DelayTreatment, 4)1**			0.24 (0.05, 1.04)	0.06
**ns(DelayTreatment, 4)2**			5.68 (0.86, 41.21)	0.08
**ns(DelayTreatment, 4)3**			0.43 (0.01, 15.93)	0.65
**ns(DelayTreatment, 4)4**			0.12 (0.00, 1.61)	0.14

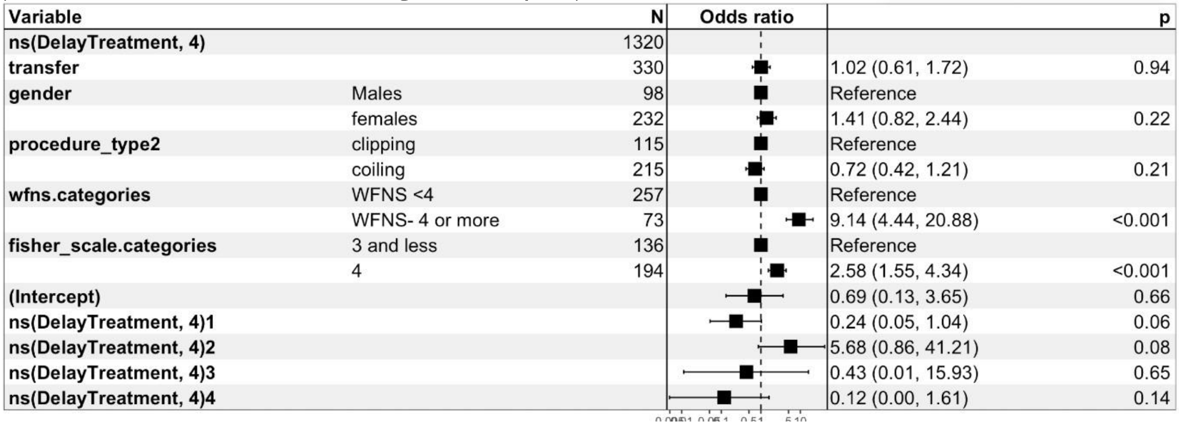

© 2022 Buscot MJ et al. *JAMA Network Open.*

eReferences

1. Baltsavias GS, Byrne JV, Halsey J, Coley SC, Sohn MJ, Molyneux AJ. Effects of timing of coil embolization after aneurysmal subarachnoid hemorrhage on procedural morbidity and outcomes. *Neurosurgery.* 2000;47(6):1320-1329; discussion 1329-1331.
2. Sarrafzadeh A, Haux D, Küchler I, Lanksch WR, Unterberg AW. Poor-grade aneurysmal subarachnoid hemorrhage: relationship of cerebral metabolism to outcome. *J Neurosurg.* 2004;100(3):400-406.
3. Frontera JA, Claassen J, Schmidt JM, et al. PREDICTION OF SYMPTOMATIC VASOSPASM AFTER SUBARACHNOID HEMORRHAGE: THE MODIFIED FISHER SCALE. *Neurosurgery.* 2006;59(1):21-27.

© 2022 Buscot MJ et al. *JAMA Network Open.*
